# Effect of host-mimicking medium and biofilm growth on the ability of colistin to kill *Pseudomonas aeruginosa*

**DOI:** 10.1099/mic.0.000995

**Published:** 2020-11-30

**Authors:** Esther Sweeney, Akshay Sabnis, Andrew M. Edwards, Freya Harrison

**Affiliations:** ^1^​ School of Life Sciences, Gibbet Hill Campus, University of Warwick, Coventry CV4 7AL, UK; ^2^​ MRC Centre for Molecular Bacteriology and Infection, Imperial College London, Armstrong Rd, London SW7 2AZ, UK

**Keywords:** antibiotics, antibiotic tolerance, biofilm, cystic fibrosis, infection models

## Abstract

*In vivo* biofilms cause recalcitrant infections with extensive and unpredictable antibiotic tolerance. Here, we demonstrate increased tolerance of colistin by *Pseudomonas aeruginosa* when grown in medium that mimics cystic fibrosis (CF) sputum versus standard medium in *in vitro* biofilm assays, and drastically increased tolerance when grown in an *ex vivo* CF model versus the *in vitro* assay. We used colistin conjugated to the fluorescent dye BODIPY to assess the penetration of the antibiotic into *ex vivo* biofilms and showed that poor penetration partly explains the high doses of drug necessary to kill bacteria in these biofilms. The ability of antibiotics to penetrate the biofilm matrix is key to their clinical success, but hard to measure. Our results demonstrate both the importance of reduced entry into the matrix in *in vivo*-like biofilm, and the tractability of using a fluorescent tag and benchtop fluorimeter to assess antibiotic entry into biofilms. This method could be a relatively quick, cheap and useful addition to diagnostic and drug development pipelines, allowing the assessment of drug entry into biofilms, in *in vivo*-like conditions, prior to more detailed tests of biofilm killing.

## Data summary

The authors confirm that all supporting data, code and protocols have been provided within the article or through the supplementary data file (Document S1).

## Introduction

Biofilm infections of host tissues or indwelling medical devices impose a significant health and economic burden, due to the high tolerance of biofilm bacteria to host immune attack and to antibiotics. Biofilm antibiotic tolerance is a function of environmentally cued changes in bacterial physiology and gene expression, and reduced penetration of some antibiotic molecules through the biofilm matrix [1]. In the case of cystic fibrosis (CF) lung disease, plugging of small airways by aggregates of biofilm embedded in abnormal host mucus leads to reduced airflow and bronchiectasis [2, 3]. In a recent analysis, bacterial lung infection was the strongest predictor of medication costs in CF, adding on average €3.6K per patient per year to direct healthcare costs [4, 5]. Intravenous antibiotic treatments, usually administered during acute exacerbations of respiratory symptoms, impose a particularly heavy burden: in the UK, people with CF spend a median of 27 days/year receiving IV antibiotics, often as hospital in-patients [6]. There is a narrow choice of antibiotics suitable for administration in CF, and a poor concordance between antibiotic susceptibility testing in diagnostic laboratories and patient outcome – even when standard *in vitro* biofilm platforms are employed for testing [7, 8].

Generic *in vitro* biofilm models (e.g. Calgary device [9, 10], flow cells) and standard laboratory growth media are usually used to test the efficacy of antibacterial agents, both in a diagnostic setting and in research and development pipelines for new agents. It is increasingly recognized that the key to optimizing biofilm management is a more context-specific approach to mimicking the in-host conditions in the infection(s) of interest. The effect of physicochemical environment, and especially of host mimicking versus standard laboratory media, on bacterial responses to antibiotics is well known [11–14]. Further, environmental differences between specific biofilm contexts are likely to produce different biofilm architectures; overall biofilm thickness and 3D structure, as well as the production of matrix polymers with different size, charge or hydrophobicity, will impact on how easily different antibiotics can penetrate the matrix to reach cells [15]. The benefits and shortcomings of different *in vitro* and *in vivo* biofilm models, and limitations on how well standard models such as the Calgary device represent *in vivo* biofilms, have been reviewed in detail by other authors [16–18].

We hypothesized that poor penetration of the *in vivo* matrix partly explains the high doses of antibiotic necessary to kill *Pseudomonas aeruginosa* in CF lung biofilms. We focused on the antibiotic colistin for two reasons. First, colistin is widely prescribed for *P. aeruginosa* infection in CF and is commonly administered by inhalation; this means that topical laboratory exposure of biofilms to colistin, by spiking the surrounding culture medium, likely mimics *in vivo* exposure better than in the case of antibiotics that are only administered orally or through IV. Second, fluorescently labelled colistin was already available in one of our laboratories, opening up the possibility to assay colistin concentration in biofilm via fluorimetry [19]. Finally, colistin is able to bind the *P. aeruginosa* exopolymer Psl [20] and extracellular lipopolysaccharide [21] and may adsorb to outer-membrane vesicles [22] – all of which may be present in *P. aeruginosa* biofilms, depending on strain and culture conditions.

We first compared the tolerance of the laboratory strain PA14 and eight CF isolates of *P. aeruginosa* to colistin in standard antibiotic susceptibility testing medium (cation-adjusted Müller–Hinton broth, caMHB) and synthetic CF sputum medium (SCFM [23]) using planktonic microdilution and biofilm eradication assays in a Calgary device. Use of SCFM led to increased minimum inhibitory concentration (MIC) in microdilution experiments for all strains, and to an increased minimum biofilm eradication concentration (MBEC) for eight of the nine strains (one showed no change). We then grew PA14 and four of the CF isolates in an *ex vivo* model of CF biofilm that combines SCFM and pig bronchiolar tissue [24, 25]. Biofilms grown in this model showed drastically increased colistin tolerance as compared with biofilms grown in the Calgary device. To test the hypothesis that this increase in tolerance is at least in part due to low penetration of the biofilm matrix by colistin, we exposed *ex vivo* porcine lung model (EVPL)-grown biofilms to fluorescently labelled colistin to measure the percentage of a dose that was able to enter the biofilm. The amount of labelled colistin present in biofilms ranged from 12–19 % of the total amount recovered from biofilms plus surrounding SCFM, demonstrating poor penetration of colistin into the biofilm matrix.

Our results confirm that the *in vivo* biofilm can prevent free diffusion of colistin to bacterial cells, and underline the limitations of using simple *in vitro* biofilm models in which bacteria are attached to an inorganic substrate and/or grown in medium that does not recapitulate the chemistry of the *in vivo* environment. The EVPL+SCFM model represents an improved balance between accurate modelling of the CF lung environment and tractability in the laboratory. We propose that it could be used to aid antibiotic susceptibility testing in diagnostic and drug development contexts. Furthermore, EVPL could be combined with fluorescent labelling of in-use or novel antibiofilm agents to produce a cheap and simple method for assessing how well these molecules enter the biofilm matrix. This could be valuable in the search for new and improved antibiofilm agents.

## Methods

### Bacterial strains

The laboratory strains PAO1 (Nottingham isolate) PA14 (a gift from Leo Eberl) and eight isolates of *P. aeruginosa* from a chronically colonized person with CF (SED6, SED7, SED8, SED9, SED11, SED13, SED17, SED19 [26]) were used in this work. The CF strains had previously been shown to vary considerably in antibiotic resistance profile, and in biofilm-forming ability as measured by crystal violet staining following growth in lysogeny broth (LB) in a Calgary biofilm device (S. Darch, personal communication) Colonies of frozen stocks were obtained by growth on LB agar at 37 °C for 18–24 h (PAO1, PA14) or 48 h (CF isolates).

### Synthetic cystic fibrosis sputum medium (SCFM)

SCFM was made following the recipe of Palmer *et al*. [23], with the modification that glucose was removed because previous work suggested that this promoted the growth of endogenous bacteria present on pig lung tissue. Even when glucose is present in SCFM, *P. aeruginosa* preferentially uses amino acids and short-chain fatty acids as carbon sources, and a recent transcriptomic study shows that this is a good reflection of *P. aeruginosa* metabolism in CF *in vivo* [23, 27].

### MIC and MBEC testing

The MIC and minimum biofilm eradication concentration (MBEC) of colistin (Acros organics) for the strains were assayed by Warwick’s Antimicrobial Screening Facility following Clinical and Laboratory Standards Institute (CLSI guidelines) in cation-adjusted Müller–Hinton broth and in SCFM. MIC testing was performed according to CLSI guidelines M7-A9 (Methods for Bacteria that Grow Aerobically), M24-A (Testing of Mycobacteria, Nocardiae, and Other Aerobic Actinomycetes) and M100-S24 (Performance Standards for Antimicrobial Susceptibility Testing). MBEC testing used the Calgary biofilm device (peg lid assay) and the methods published by Moskowitz *et al.* [10].

### EVPL

The EVPL was prepared as described previously [24, 25], except that SCFM was not supplemented with antibiotics to repress the growth of any endogenous bacteria present on the lung tissue: this was to prevent any interference with assessment of colistin efficacy in later experiments. *P. aeruginosa* has been shown to produce CF-like biofilm in this model [24].

Pig lungs were obtained from Quigley and Sons, Cubbington, and John Taylor and Son, Earlsdon, and dissected on the day of delivery under sterile conditions. The pleura of the ventral surface was heat-sterilized using a hot pallet knife. A sterile razor blade was then used to make an incision in the lung, exposing the bronchiole. A section of the bronchiole was extracted and the exterior alveolar tissue removed using dissection scissors. Bronchiolar sections were washed in a 1 : 1 mix of Dulbecco’s modified Eagle’s medium (DMEM) and RPMI 1640 supplemented with 50 µg ml^−1^ ampicillin (Sigma-Aldrich). Bronchioles were then cut into squares, with a further two washes in DMEM+RPMI+ampicillin during dissection. The bronchiole squares were then washed twice in SCFM (no antibiotics), UV-sterilized for 5 min in a germicidal cabinet and transferred to individual wells of a 24-well plate containing 400 µl SCFM solidified with 0.8 % (w/v) agarose per well. Tissue sections were inoculated by touching a sterile 29G hypodermic needle (Becton Dickinson Medical) to the surface of a *P. aeruginosa* colony on LB agar, and gently piercing the surface of the tissue section. Uninfected control sections were mock inoculated with a sterile needle. Five hundred microlitres of SCFM was added to each well. Plates were sealed with Breathe-Easier gas-permeable membrane (Diversified Biotech) and incubated at 37 °C for the desired length of time.

### Assessing repeatability of *P. aeruginosa* biofilm load on *ex vivo* pig bronchiole

Eighteen bronchiolar sections from each of three sets of pig lungs were prepared as above. Twenty-four-well plates containing tissues were photographed using a 20 MP digital camera placed at a set height on a copy stand and these images were later used to calculate the surface area of each tissue section using the freehand select tool in ImageJ: as we dissect tissues by hand we wanted to determine whether variation in section size affected bacterial load. The mean size of tissue section was 44 mm^2^ (sd 11.5 mm^2^). Six sections of tissue were inoculated with PA14 and six with PAO1; the remaining six were left uninoculated. After 2 and 7 days incubation at 37 °C, half of the tissue sections plus associated biofilm were removed, briefly washed in 500 µl PBS in a fresh 24-well plate to remove loosely adhering planktonic cells, and then placed into 1 ml PBS in screw-cap homogenization tubes (Fisherbrand) containing 18 2.38 mm metal beads (Fisherbrand). Tissue was bead-beaten in a FastPrep-24 5G (MP Biomedicals) for 40 s at 4 m s^−1^ to recover the bacteria from the tissue-associated biofilm. Bacterial load was calculated by serially diluting the homogenates, plating on LB agar and counting colony-forming units (c.f.u.).

### Production of labelled colistin

This process is described in detail by Sabnis *et al.* [19]. Briefly, colistin was labelled by incubation with BODIPY FL SE D2184 (Thermo Fisher Scientific) and sodium bicarbonate for 2 h at 37 °C; unbound BODIPY was removed by dialysis; and successful labelling was confirmed by time-of-flight mass spectrometry.

### The effect of colistin on EVPL-grown biofilms

PA14 and clinical isolates SED6, SED8, SED17 and SED19 were cultured in EVPL+SCFM for 2 days, and tissue pieces plus surrounding biofilm were then transferred to new 24-well plates containing 1 ml fresh SCFM+colistin at 0.5× *in vitro* MIC in SCFM, 4× *in vitro* MIC in SCFM, 0.5× Calgary device MBEC in SCFM, 4× Calgary device MBEC in SCFM and 10× Calgary device MBEC in SCFM, or antibiotic-free SCFM (three pieces of tissue per strain, per treatment). The tissue sections with associated biofilms were incubated in these treatment plates for 18 h at 37 °C, and then bacteria were recovered, bead-beaten as above and diluted and plated on LB agar for enumeration of viable c.f.u.

### Treatment of EVPL biofilms with BODIPY/colistin

The addition of the BODIPY-tag has been shown to reduce bactericidal activity in planktonic culture (MIC in microdilution assay in MHB increased from 0.5 µg ml^−1^ to 1 µg ml^−1^ [19]). Thus, concentrations of BODIPY/colistin that correspond to sub-inhibitory and bactericidal concentrations of unlabelled colistin were chosen from inspection of the data on EVPL biofilms treated with unlabelled colistin. Following CLSI recommendations (M26-A, Methods for Determining Bactericidal Activity of Antimicrobial Agents) the minimum bactericidal concentration (MBC) of colistin in the EVPL was defined as the lowest concentration causing a ≥3-log_10_ reduction in median c.f.u. recovered from biofilms, compared with untreated biofilms. These concentrations were chosen as ‘bactericidal’ concentrations of BODIPY/colistin to use in fluorescent colistin penetration assays. We chose 2 µg ml^−1^ as the sub-inhibitory concentration for later work with PA14, and 8 µg ml^−1^ as the sub-inhibitory concentration for later work with all CF isolates.

PA14, SED6, SED8, SED17 and SED19 were inoculated into replica pieces of EVPL and incubated for 2 days at 37 °C to form mature biofilm, and tissue pieces were then transferred individually to 30 ml soda glass screw-top vials containing 1 ml fresh SCFM alone or 1 ml SCFM+colistin labelled with BODIPY FL SE D2184 (succinimidyl ester; Thermo Fisher Scientific) at concentrations corresponding to sub-inhibitory or bactericidal concentrations of unlabelled colistin. Three uninfected pieces of EVPL were incubated alongside the infected tissue sections and transferred individually to 30 ml soda glass screw-top vials containing 1 ml fresh SCFM alone. Labelled colistin was produced as described previously [19]. Tubes were incubated for 18 h at 37 °C. Glass vials were used for these experiments because colistin can bind plastic and we wished to minimize loss to binding of the culture vessel in order to measure the proportion of colistin entering the biofilms as accurately as possible. A pilot experiment in which BODIPY/colistin exposure was conducted in plastic 24-well plates (Corning Costar) resulted in only ~50 % of the original BODIPY/colistin dose being recovered at the end of the treatment period, consistent with colistin binding plastic (data not shown).

Tissue with associated biofilm was removed from the glass tubes and homogenized in 1 ml SCFM. Replica aliquots of this homogenate were used for c.f.u. plating. BODIPY/colistin was immediately quantified in 100 µl aliquots of tissue+biofilm homogenate and surrounding SCFM by fluorimetry (100 µl in a black 96-well plate with ex/em 485/535 nm in a Tecan SPARK 10M multimode plate reader). There was slightly more background fluorescence signal from infected but untreated EVPL sections than from uninfected untreated EVPL (see Document S1, available in the online version of this article), therefore fluorescence values from samples exposed to BODIPY/colistin were standardized by subtracting the mean fluorescence measured in homogenate/surrounding ASM from tissues infected by the same strain but exposed to SCFM with no BODIPY/colistin. A calibration curve using BODIPY/colistin in SCFM (Fig. S5) was used to calculate total amounts of BODIPY/colistin in the whole biofilm and the whole volume of surrounding SCFM. The percentage of the original dose of BODIPY/colistin recovered from both biofilm and SCFM combined was then calculated, to determine if any of the dose had been lost due to binding the glass, lung tissue and/or homogenization beads.

## Results

### Effect of SCFM on MIC and MBEC of colistin

**Fig. 1.**
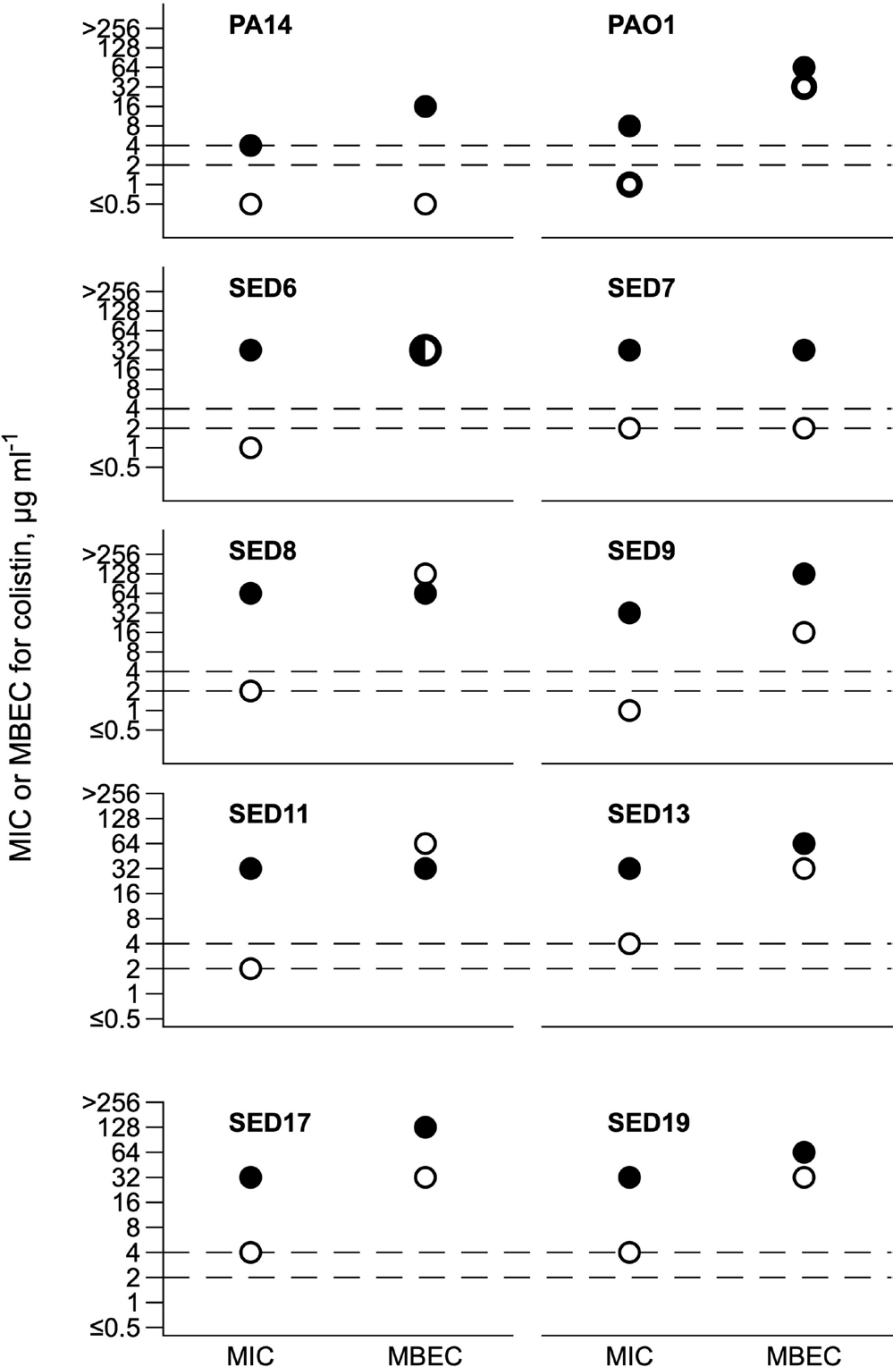
MIC by broth microdilution and MBEC in Calgary biofilm device of colistin for PA14, PAO1 and eight CF isolates of *P. aeruginosa *. Open symbols show values for tests conducted in cation-adjusted Müller–Hinton broth, closed symbols show values for tests conducted in synthetic CF sputum medium; note that for SED 8, the MBEC was the same in both media. The dashed lines denote the CLSI breakpoints for classification as sensitive (≤2 µg ml^−1^) or resistant (≥4 µg ml^−1^) in MIC testing in cation-adjusted Müller–Hinton broth. Full data are supplied in Document S1; MIC values are taken from two replica assays and MBECs are from a single assay.

As expected, the inhibitory concentration of colistin varied depending on culture medium (SCFM usually >caMHB) and growth mode (MBEC >MIC in all cases but one) (Fig. 1). For 7 of the 10 isolates, conducting MIC testing in SCFM instead of caMHB led to a change in classification from sensitive to resistant. We selected CF isolates SED6, SED8, SED17 and SED19 for further work with the EVPL, as these had a range of MIC/MBEC values (MIC 1–4 µg ml^−1^ in caMHB and 32–64 µg ml^−1^ in SCFM; MBEC 32–128 µg ml^−1^ in both media) and included two isolates for which MBEC=MIC in SCFM (SED6, SED8) and two for which MBEC >MIC in SCFM (SED17, SED19).

### Reproducibility of biofilm load associated with tissue in EVPL

Reproducibility of biofilm load is essential in any model system that is intended for use in the testing of antibiofilm or antibacterial agents. We therefore assessed the reproducibility of biofilm load between pieces of tissue from the same/different lungs by measuring the c.f.u. (by plating on LB agar) in biofilms of PAO1 and PA14 at 2 and 7 days post-infection (p.i.). We wished first to address two questions. First, whether there was a statistically significant interaction between lung and *P. aeruginosa* strain in determining total c.f.u. or c.f.u. mm^−2^, i.e. did any difference between PA14 and PAO1 depend on whether they were in tissue from lungs 1, 2 or 3? A lack of such an interaction would suggest that experimental results are robust to any natural variation in tissue between lungs taken from different animals. Second, we wished to calculate the statistical repeatability of total c.f.u. or c.f.u. mm^−2^ across lungs 1–3 for each of the two strains as a measure of reproducibility. Based on the answers to these two questions, we then aimed to make decision about (1) whether future experiments should measure the effects of antibiotics in terms of total c.f.u. or whether we should standardize for variation in the size of hand-cut tissue sections by working with counts of c.f.u. mm^−2^; and (2) whether one of the two laboratory strains grew more consistently in the model and thus would be better to use as a standard ‘wild-type’ in future work.

Fig. S1 shows photographs of tissues at inoculation and at 2 and 7 days p.i.; *P. aeruginosa* growth is clearly visible due to the typical blue-green pigmentation of this species. All colonies recovered from tissue infected with *P. aeruginosa* were identified as *P. aeruginosa* by morphology; uninfected tissues cultured a high load of endogenous bacteria from the lungs, but these were clearly distinguishable from *P. aeruginosa* by size, shape and colour (* P. aeruginosa* colonies had a characteristic blue-green colour with a slightly fuzzy margin on LB agar, even after growth in the EVPL, whereas endogenous bacteria cultured from the uninfected lungs were all white or pale yellow with a lenticular appearance). As shown in Fig. S2, both PAO1 and PA14 reached biofilm loads comparable with those reported for CF (sputum microbiology studies routinely give upper limits on the detected *P. aeruginosa* load of 10^7^–10^10^ c.f.u. ml^−1^ [28–30] and *P. aeruginosa* aggregates, suggestive of detached biofilm fragments, are observable in CF sputum [2, 31, 32]). As detailed in Fig. S3 and Table S1, PA14 showed more reproducible biofilm loads than PAO1; biofilm loads were more repeatable at day 2 than day 7. Standardizing c.f.u. by tissue area (measured in mm^2^ using ImageJ, [33]) did not improve repeatability, given the variation inherent in cutting tissue by hand (see Document S1), this result was initially surprising. However, the tissue may simply be a scaffold, or cue, for physiologically realistic biofilm formation and maturation, rather than a nutrient source. If bacteria gain most of their nutrients from the ASM rather than the tissue, attempting to standardize by tissue area is unnecessary – the bacteria have reached their carrying capacity, so dividing this by area, which varies considerably, adds stochastic variation. Based on these preliminary results, we decided to use PA14 as an example laboratory isolate in further work; to grow biofilms for 2 days prior to colistin treatment; and not to standardize c.f.u. counts by tissue area when measuring viable cells in biofilms.

### Biofilms of *P. aeruginosa* grown in EVPL have drastically increased tolerance to colistin

**Fig. 2.**
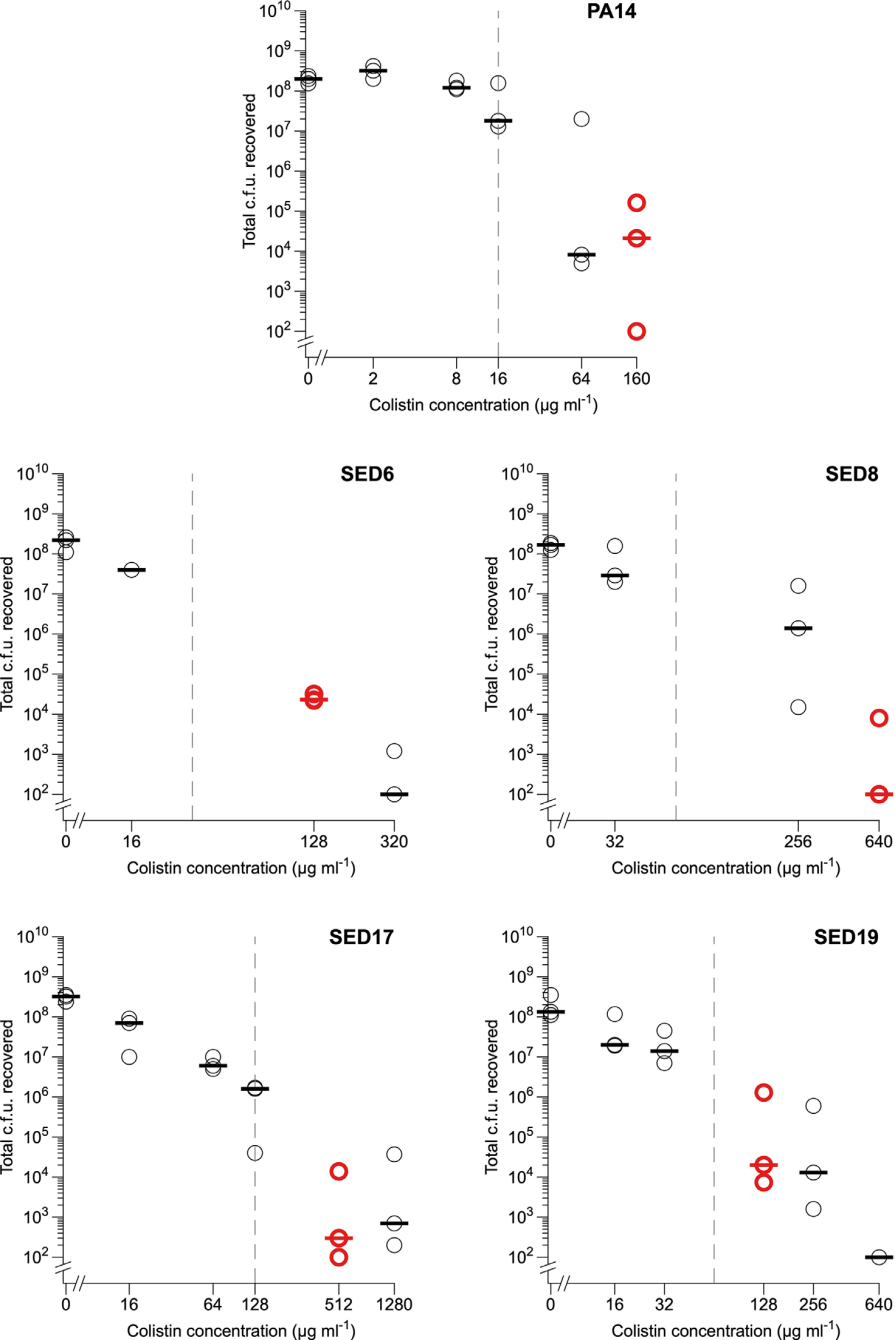
Effect of treating 2-day EVPL biofilms of *P. aeruginosa* with colistin for 18 h. Each data point represents a single tissue section, and all tissue sections were taken from the same pair of pig lungs. Horizontal lines denote medians. Red points show the lowest concentration of colistin causing a ≥3-log_10_ reduction in c.f.u. compared with untreated biofilms (an outlier for PA14 at 64 µg ml^−1^ was discounted). PA14, SED17 and SED19 had differing values for broth microdilution MIC and Calgary device MBEC tests in SCFM, and so were treated with colistin at 0.5× *in vitro* MIC, 4× *in vitro* MIC, 0.5× Calgary device MBEC, 4× Calgary device MBEC and 10× Calgary device MBEC. SED6 and SED8 had equal broth microdilution MIC and Calgary device MBEC values in SCFM and so were treated with colistin at 0.5× *in vitro* MIC/Calgary device MBEC, 4× *in vitro* MIC/Calgary device MBEC and 10× *in vitro* MIC/Calgary device MBEC. Grey dashed lines show each isolate’s MBEC in SCFM in the Calgary device. Note that tissues+biofilms were exposed to BODIPY/colistin in a total volume of 1 ml SCFM, therefore concentrations correspond to total µg present. Raw data, R code and results of ANOVA analyses of c.f.u. in untreated biofilms formed by each strain are supplied in Document S1.

We then aimed to assess the bactericidal activity of colistin to EVPL-grown *P. aeruginosa *, and to identify concentrations that were sub-inhibitory or bactericidal to these biofilms for use in later experiments with labelled colistin. PA14 and clinical isolates SED6, SED8, SED17 and SED19 were cultured in EVPL+SCFM for 2 days and the bronchiolar section, plus associated biofilm, was transferred to fresh SCFM containing colistin at 0.5× *in vitro* MIC in SCFM, 4× *in vitro* MIC in SCFM, 0.5× Calgary device MBEC in SCFM, 4× Calgary device MBEC in SCFM or 10× Calgary device MBEC in SCFM, or antibiotic-free SCFM (three pieces of tissue per strain, per treatment). The tissue sections and associated biofilm were incubated in the colistin treatment for 18 h at 37 °C, and bacteria were recovered for the enumeration of viable c.f.u. The results in Fig. 2 show that growth as biofilms in EVPL leads to a drastic increase in colistin tolerance compared with growth in *in vitro* surface-attached biofilm models, even when SCFM was used in conjunction with the *in vitro* MBEC platform. This is potentially due to decreased bacterial sensitivity, low penetration of colistin into the biofilm matrix and/or colistin-binding proteins in the bronchiolar tissue [34]

Following CLSI recommendations (M26-A, Methods for Determining Bactericidal Activity of Antimicrobial Agents) the MBC of colistin in the EVPL was defined as the lowest concentration causing a ≥3-log_10_ reduction in median c.f.u. recovered from biofilms, compared with untreated biofilms (red circles in Fig. 2). These concentrations were chosen as ‘bactericidal’ concentrations of BODIPY/colistin to use in fluorescent colistin penetration assays (note that addition of BODIPY to colistin reduces its activity as an antibiotic). This corresponded to 10× the ASM MBEC for PA14 and SED8, 4× the ASM MBEC for SED6 and SED17, and 2× the SCFM MBEC for SED19. MBCs differed fivefold between the strains used. This is likely due to differences in biofilm matrix structure or other genetic differences between the isolates, as all grew to similar cell densities in the absence of colistin [analysis of variance (ANOVA) for effect of strain identity on *P. aeruginosa* c.f.u. in untreated biofilms only: F_4,10_=1.49, *P*=0.277]. We chose 2 µg ml^−1^ as the sub-inhibitory concentration for later work with PA14, and 8 µg ml^−1^ as the sub-inhibitory concentration for later work with all CF isolates.

### BODIPY-tagged colistin is poorly able to enter EVPL-grown biofilms

PA14, SED6, SED8, SED17 and SED19 were inoculated into replica pieces of EVPL, incubated for 2 days at 37 °C to form mature biofilm, and tissue sections+associated biofilm were then transferred individually to the wells of 24-well culture plates containing 1 ml fresh SCFM alone or 1 ml SCFM containing BODIPY-tagged colistin at concentrations corresponding to sub-inhibitory (2 µg ml^−1^ for PA14, 8 µg ml^−1^ for the CF isolates) or bactericidal concentrations (see Fig. 2) of unlabelled colistin. Three uninfected pieces of EVPL were incubated alongside the infected tissue sections, and transferred individually to 1 ml fresh SCFM with no colistin. The tissue and associated biofilm was incubated in the BODIPY/colistin treatment for 18 h at 37 °C. A pilot experiment using PA14, SED6 and SED8 had confirmed that, as in planktonic culture [19], the BODIPY tag significantly reduced the antibacterial activity of colistin in this biofilm assay (Fig. S3).

At the end of the 18 h exposure to BODIPY/colistin, the tissue sections were removed from the treatment and biofilms were recovered by bead-beating the individual tissue sections in 1 ml SCFM. Replica aliquots of the biofilm homogenate were used for c.f.u. plating and for quantification of BODIPY/colistin. Aliquots of the SCFM surrounding tissue sections in the treatment plate were also removed for quantification of BODIPY/colistin. BODIPY/colistin quantification was performed by fluorimetry in a Tecan SPARK 10M multimode plate reader. A calibration curve of BODIPY/colistin added to SCFM and left at 37 °C for 18 h (Fig. S4) was used to calculate the total amount of BODIPY/colistin in each biofilm and in the SCFM surrounding it. The percentage of the original dose of BODIPY/colistin recovered from both biofilm and SCFM combined was then calculated to determine if any of the dose had been lost due to binding the glass, lung tissue and/or homogenization beads.

The recovery rates of total BODIPY/colistin from the biofilm homogenate plus surrounding SCFM were fairly good for medium–high concentrations, peaking at 93–100 % recovery from lung sections receiving a dose of 128 µg ml^−1^ (Fig. S5). At lower concentrations, recovery rates dropped, and became very variable at the lowest dose of 2 µg ml^−1^ (used for PA14). This means that the values for this specific treatment may be unreliable. Our recovery rate of 70–80 % at the second lowest dose (8 µg ml^−1^) was consistent with recovery rates in soda glass reported by a previous study, which compared the loss of colistin to substrate binding in various materials [35]. Better rates were achieved for higher concentrations, although recovery did then start to tail off as concentrations became very high.

**Fig. 3.**
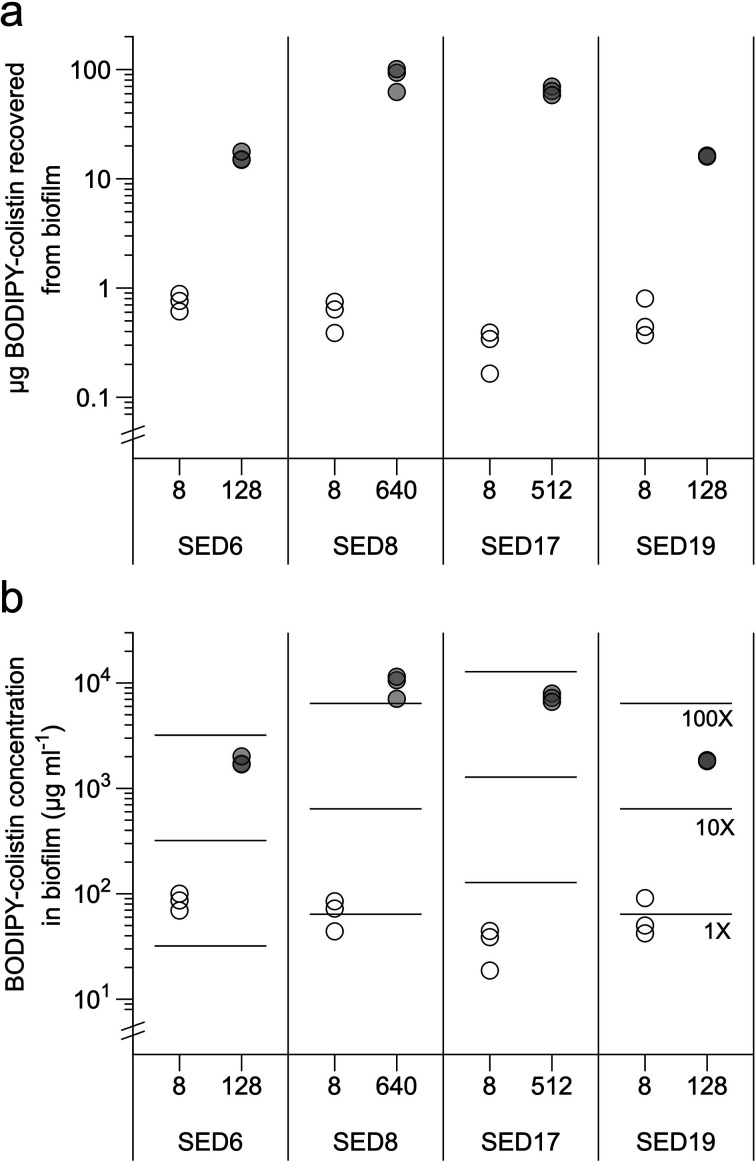
Amount of BODIPY/colistin present in biofilms of CF isolates after 18 h exposure, as measured by fluorimetry of biofilm homogenate. Each symbol is one tissue section, numbers on the *x*-axis are the sub-inhibitory and MBC exposure doses used. (a) BODIPY/colistin measured (µg). (b) Concentration of BODIPY/colistin in biofilm, assuming a biofilm volume of 8.8 µl. For reference, the lines show 1, 10 and 100× each strain’s MBEC as measured in the Calgary biofilm device using SCFM. Raw data, and data for PA14, are supplied in Document S1.

Fig. 3a shows the amount of BODIPY/colistin measured in the tissue-associated biofilms for the CF isolates; PA14 is not shown due to the BODIPY quantification becoming unreliable in the 2 µg ml^−1^ treatment (data are supplied in Dcument S1). The amount of BODIPY/colistin in biofilms ranged from 12–19 % of the total amount recovered from biofilms+surrounding SCFM (means and standard deviations: SED6 13±1 %, SED8 16±4 %, SED17 15±2 %, SED19 13±0 %). To calculate the concentration of BODIPY/colistin present in biofilms, the volume of the biofilm was estimated. Conservative estimation of this concentration is achieved by estimating the largest possible biofilm present on the tissue. Assuming complete coverage of both sides of a 44 mm^2^ tissue section (the average area, see above) by biofilm 100 µm deep (as measured for PA14 in another study using this model, [24]), this suggests that a sensible maximum biofilm volume to use is 8.8 µl. Fig. 3b shows the predicted BODIPY/colistin concentrations in the biofilms assuming this volume. The concentrations of BODIPY/colistin inside the biofilm following supplementation of the surrounding SCFM at MBC are between 10 and 100× the MBEC measured for the same strain in a Calgary biofilm device using SCFM as the culture medium. Obtaining more exact measurements of the in-biofilm concentration would require imaging work to reveal the exact size and shape of biofilms on the tissue surface (for example, two strains could form biofilms with the same c.f.u. of bacteria, but different amounts of matrix; or one strain may form a much thinner biofilm that is spread over a larger area than the other).

## Discussion

In-host environments can cue changes in bacterial transcriptome and physiology that will affect sensitivity to antibiotics (e.g. changes in membrane biology, expression of efflux pumps or beta-lactamases [11, 14, 27]). Further, the biofilm mode of growth can itself trigger physiological changes in antibiotic tolerance (e.g. antibiotics that target transcription/translation are not active against quiescent cells deep within biofilms [36]). But a big issue with *in vivo* biofilms is the inability of antibiotics to penetrate the extracellular matrix. Penetration capabilities will depend on the molecular properties of specific drugs, and on the nature of the *in vivo* biofilm matrix. Understanding how the *in vivo* matrix retards drug penetration is a key question in antibiotic choice, dosing and development.

This study shows that colistin, a drug commonly prescribed for *P. aeruginosa* lung infection in people with CF, shows very low ability to enter the biofilm matrix when this bacterium is grown as a CF-like bronchiolar biofilm. Combining SCFM with lung tissue to grow CF-like biofilms leads to tolerance of much higher concentrations of colistin than using SCFM with a standard *in vitro* biofilm platform, and this is likely a combination of changes in cellular physiology and the biofilm matrix architecture, as <20 % of the labelled colistin to which biofilms were exposed was able to enter the biofilm (these percentages take into account loss of colistin due to adsorption or binding to the lung tissue, glass exposure vial, plastic homogenization tube and/or metal homogenization beads). Peak sputum concentrations of colistin following delivery by inhalation are in the range of 1–40 µg ml^−1^, declining to 1–10 µg ml^−1^ 12 h post-dose [37, 38], well below the concentrations necessary to observe significant killing of bacteria in entrenched biofilms in our lung model. Colistin dose is limited by its nephrotoxicity. Our results underline the reasons why antibiotics cannot completely clear biofilm infection once it is established: the large doses required to penetrate and kill bacteria in large biofilm aggregates are not physiologically achievable.

EVPL is a tractable and reproducible model for growth of *in vivo*-like *P. aeruginosa* biofilms. Our experiments using EVPL and SCFM alongside standard antibiotic susceptibility testing platforms demonstrate three important microbiological results. First, we confirm that in-host diversity may be important for overall infection AMR: different *P. aeruginosa* clones taken from a single CF sputum sample have different MICs and MBECs *in vitro* in Müller–Hinton broth and SCFM, and different MBCs in the EVPL biofilm model. Second, growth as *in vivo*-like biofilm in EVPL increases colistin tolerance well beyond what is observed in the Calgary device, even when SCFM is used in place of Müller–Hinton broth. This underlines the limitations of *in vitro* models of *in vivo* biofilms. Finally, the results confirm that the *in vivo* biofilm prevents free diffusion of colistin to bacterial cells; most colistin administered remains in the SCFM surrounding the biofilm. Clinicians currently have little information beyond planktonic or agar plate MIC to support their choice or dose of antibiotic, so if the EVPL biofilm methodology could be used with microbiological samples from people with biofilm infections (e.g. expectorated sputum), it could facilitate personalized diagnostic AST with greater predictive power, and improved antibiotic stewardship.

Various formulations of artificial CF sputum or mucus are available [13, 23, 39]. We chose to work with a version designed following chemical comparison with CF sputum samples and validated by comparing the transcriptome, preferred carbon sources and quorum-sensing signalling activity of a well-characterized laboratory strain of *P. aeruginosa* in the SCFM and in CF sputum [23]. A modified version of SCFM, termed SCFM2, was later developed in the same research group. This adds bovine maxillary mucin, salmon sperm DNA, N-acetyl glucosamine and dioleoylphosphatidylcholine to the medium to further match the composition of CF sputum [39]. Crucially, colistin binds mucins [40], thus the use of SCFM without mucin allowed us to interrogate the role of bacterially produced components of the biofilm matrix in hindering access of colistin to cells. Further, we have shown that some key aspects of *P. aeruginosa* biology (total biofilm load, purine metabolism, production of quorum-sensing signal and virulence factors) do not significantly change in the EVPL model when SCFM2 is substituted for SCFM, and that extracellular DNA is present in *P. aeruginosa* biofilms grown in EVPL+SCFM [24]. Given the extra cost and time required to prepare SCFM2 (which requires sterilization of the mucin and large-scale phenol extraction of commercially sourced salmon sperm DNA prior to use), we propose that use of EVPL+SCFM represents an adequate balance between physiological relevance and accessibility for much research on *P. aeruginosa *. No model is a perfect match for *in vivo* conditions, and this model provides a platform for performing AST in a more *in vivo*-like context than standard laboratory media, while remaining cheap and straightforward to implement. While use of SCFM2 may further enhance the likeness of EVPL biofilms to *in vivo* CF biofilms, we conclude that individual researchers must make this choice based on their exact study question and local constraints.

These results also suggest that EVPL can be combined with BODIPY labelling of in-use or novel antibiofilm agents to produce a cheap and simple method for assessing how well these molecules enter the biofilm matrix. Methods such as confocal or super-resolution microscopy, or mass spectrometry-based imaging, are already used to visualize how drugs penetrate biofilms, but these require specialized and expensive equipment: this much simpler methodology could speed up biofilm efficacy screening for new antibiotics, and for adjuvants proposed to enhance biofilm entry of antibiotics. The main limitation of this approach is unreliable recovery and/or quantitation of BODIPY/colistin at the lowest exposure dose used (2 µg ml^−1^). Further optimization of the protocol may allow the use of lower concentrations of labelled molecules, or alternative approaches such as HPLC or ELISA may need to be used when working with low concentrations. A second potential limitation is our assumption of uniform biofilm coverage of the tissue in calculating biofilm concentrations of BODIPY/colistin. More detailed analysis of light/confocal microscopy images, or quantification of matrix components in biofilms produced by different strains, would be necessary to produce more accurate calculations of biofilm volume and thus BODIPY/drug concentration. The present research focuses on CF, but the combination of an *ex vivo* model using host-mimicking surfaces/media plus fluorescently tagged antibiotics could also be applied to work on other hard-to-treat biofilm infections, e.g. ventilator-associated pneumonia [41], chronic obstructive pulmonary disease [42], chronic wounds [43] or medical device infections [44].

Understanding why biofilms are so refractory to treatment and finding new anti-biofilm therapies are priorities in bacteriological research and in industrial R and D [45]. Our results underline the extent and diversity of biofilm matrix resistance to antibiotic entry in CF-like biofilm; they also provide a platform for more mechanistic exploration of the properties of the *in vivo* biofilm matrix.

## Supplementary Data

Supplementary material 1

Supplementary material 2
