# Supplementary material for: Effect of host-mimicking medium and biofilm growth on the ability of colistin to kill Pseudomonas aeruginosa
Source: Microbiology (Reading). 2020 Nov 30;166(12):1171–80. doi: 10.1099/mic.0.000995 (PMC7819359; doi:10.1099/mic.0.000995)
Supplement: Supplementary material 1 [file mic-166-1171-s001.pdf]

Supplementary Information

Effect of host-mimicking medium and biofilm growth on the ability of colistin to kill *Pseudomonas aeruginosa.*

Esther Sweeney[1^], Akshay Sabnis[2], Andrew M. Edwards[2] & Freya Harrison[1*^]

[1]School of Life Sciences, Gibbet Hill Campus, University of Warwick, Coventry CV4 7AL

[2]MRC Centre for Molecular Bacteriology and Infection, Imperial College London, Armstrong Rd., London, SW7 2AZ

*Author for correspondence: f.harrison@warwick.ac.uk

[^]These authors contributed equally to the manuscript

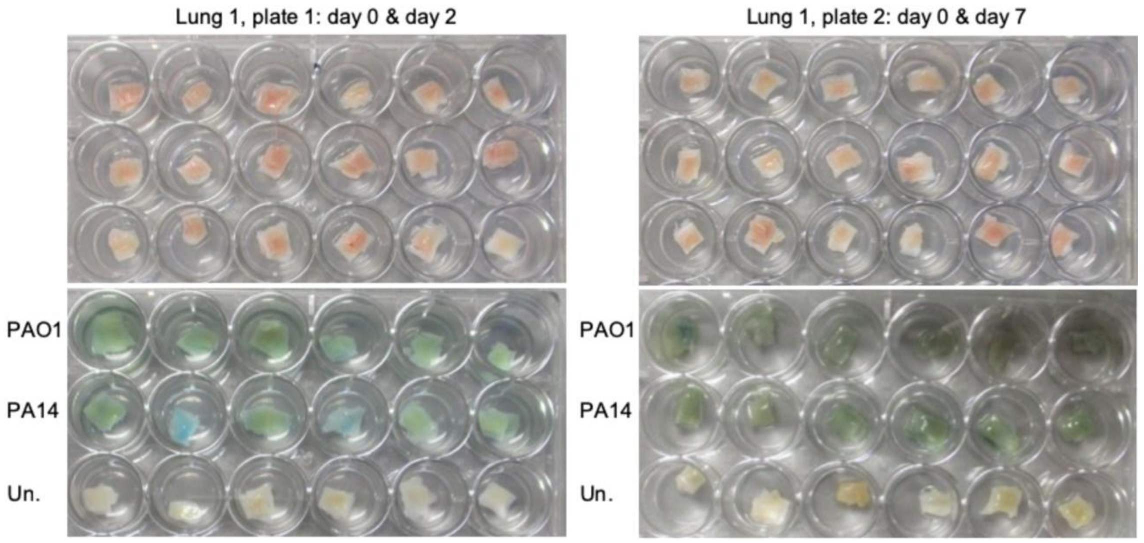

Fig. S1 Photographs of example tissue sections and biofilms. Sections of pig bronchiole from one lung are shown in standard 24-well culture plates prior to inoculation (top panels) and after either two or seven days incubation at 37°C.

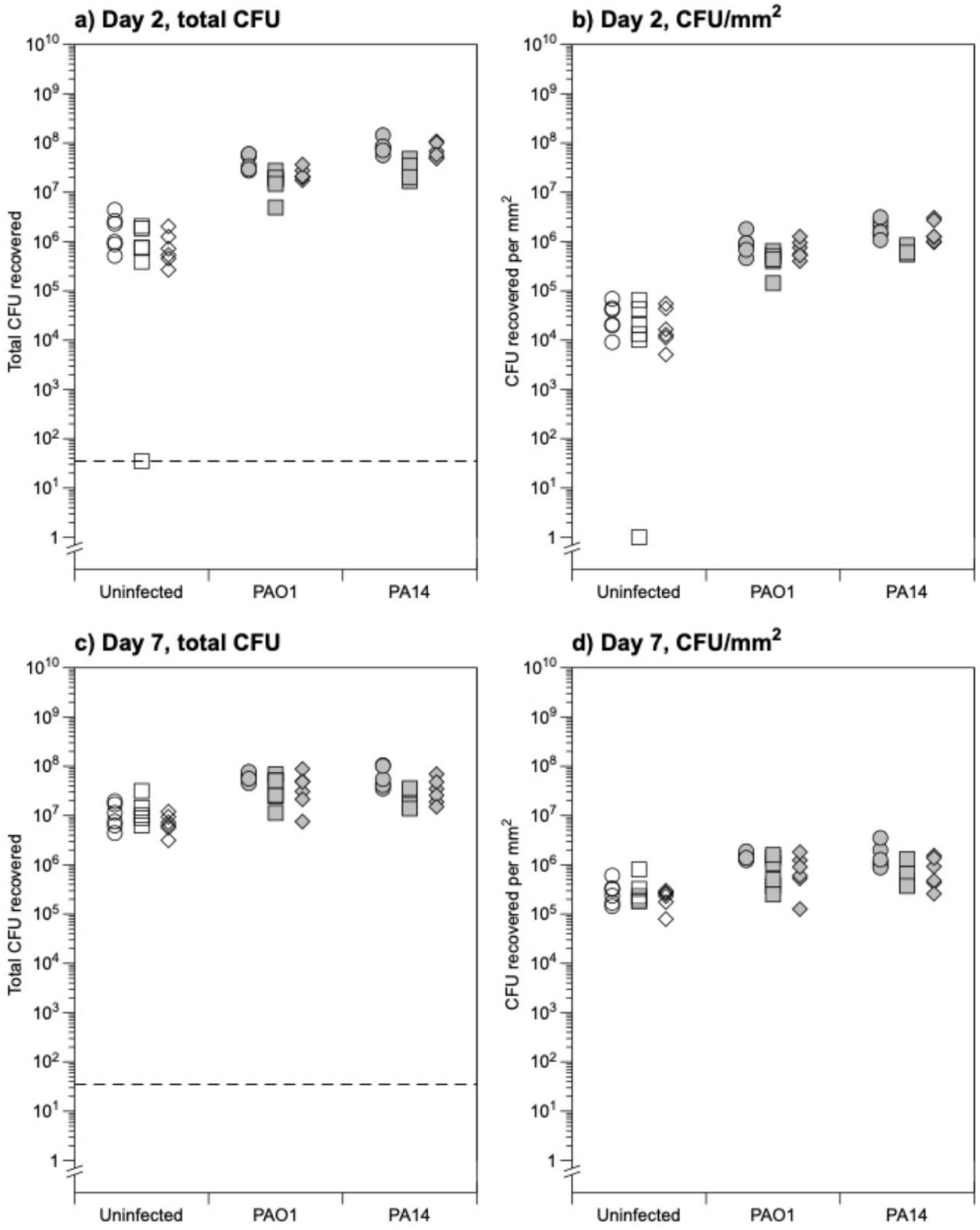

Fig. S2 Bacterial c.f.u. counts in biofilm harvested from tissue sections at 2 and 7 days post infection. Open symbols denote colonies of endogenous bacteria, closed symbols colonies identifiable as *P. aeruginosa*. Circles: lungs 1; squares: lung 2, diamonds: lung 3. ANOVA showed there was no significant interaction between lung and strain at either day, in total or area-standardised data (see Table S1). Raw data, R code and full results of ANOVA analyses of these data are supplied in Document S1.

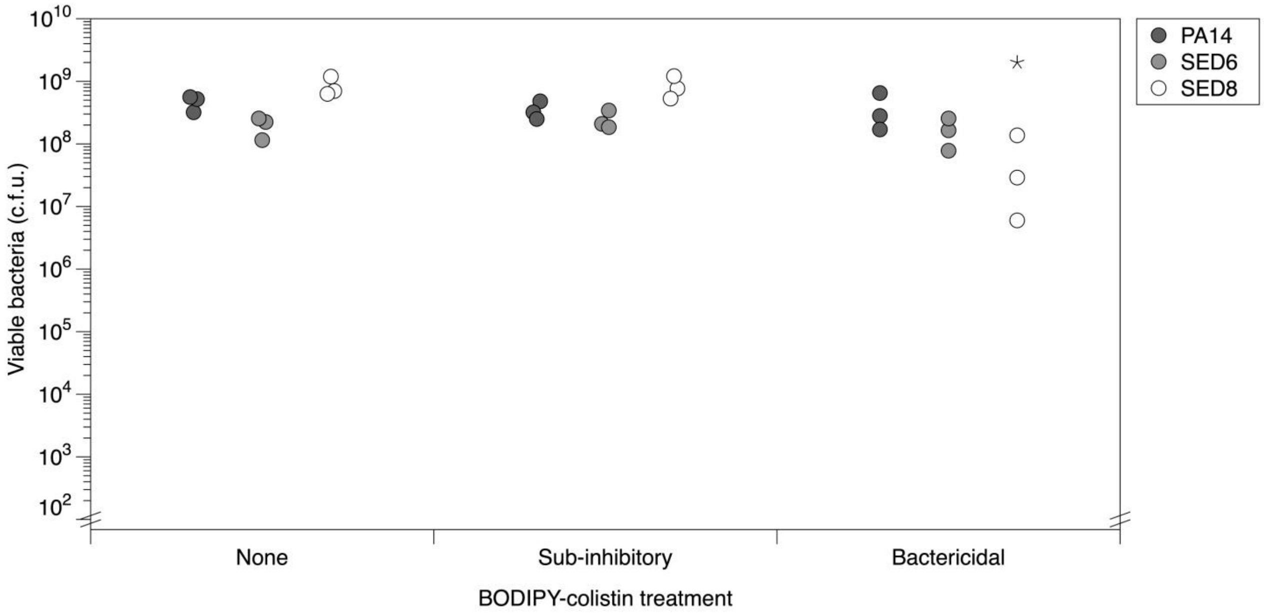

Fig. S3 Effect of BODIPY-colistin on viable cells recovered from EVPL biofilms. SED6, PA14 and SED8 were selected as examples of strains with low, medium and high (128, 160 and 640 µg ml^{-1}) bactericidal concentrations of BODIPY-colistin, respectively. Aliquots of homogenised tissue+biofilm from these strains was diluted and plated for c.f.u. counting after the exposure period. The graph shows c.f.u. recovered from individual tissue sections. ANOVA was used to test for effects of strain, treatment (no, subinhibitory or bactericidal concentration) and their interaction. The residual mean square from the ANOVA was used to conduct planned pairwise t-tests to compare the mean c.f.u. from biofilm exposed to BODIPY-colistin with the c.f.u. from biofilms of the same strain that were not treated with BODIPY-colistin. A significant drop in c.f.u. was observed only for SED8 treated with the highest concentration (denoted with *; $t_{18} = 6.23$, $p < 0.001$; c.f.u. was approx. 20% of that observed in untreated biofilms), all other comparisons were not significant. Full data and statistical analysis are supplied in Document S1.

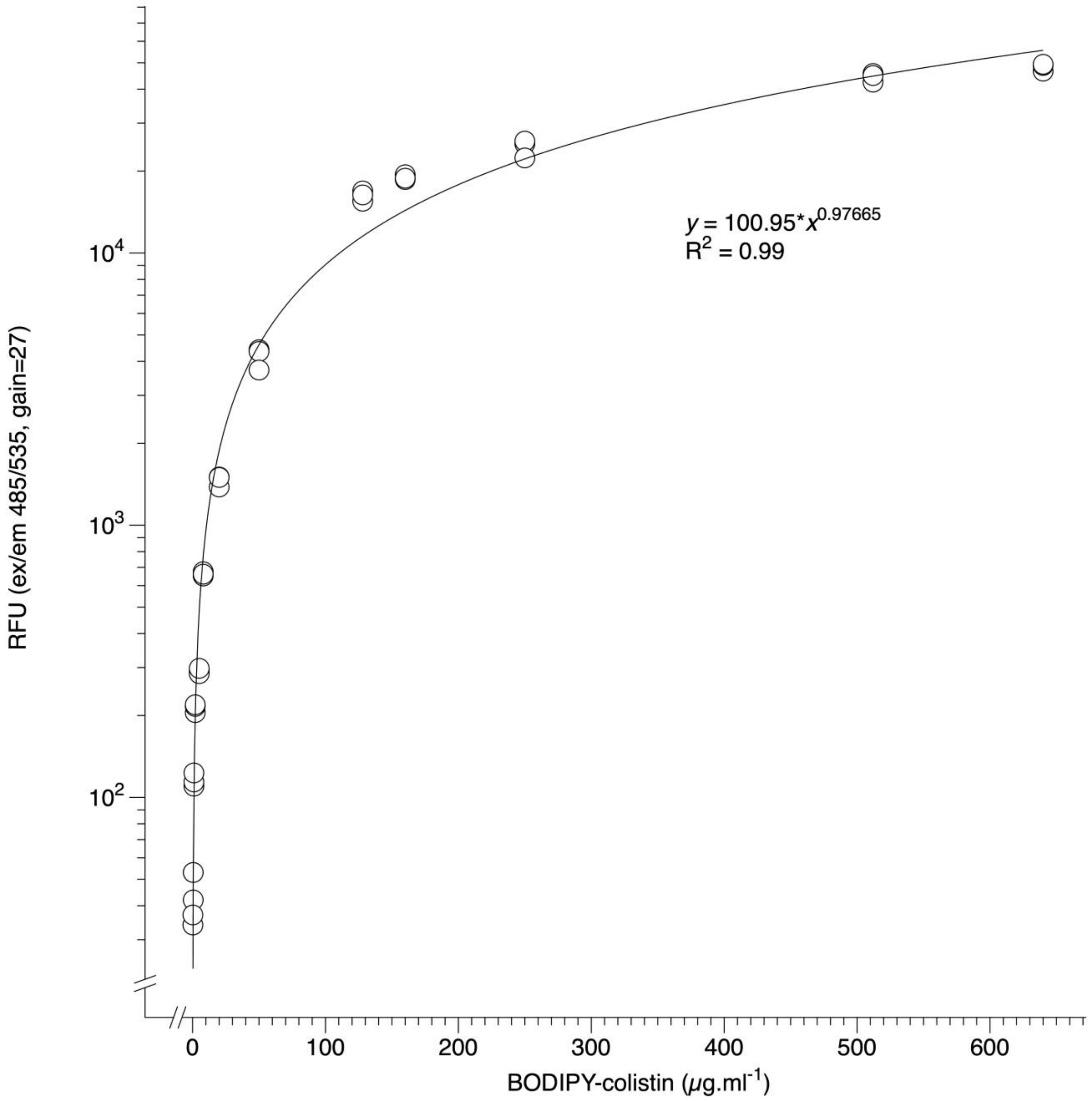

Fig. S4 Calibration curve for BODIPY-colistin fluorescence. BODIPY-colistin was diluted in SCFM (0-640 µg ml^{-1}) and incubated for 18h at 37°C before fluorescence (relative fluoresce units, RFU) was measured for triplicate 100 µl aliquots, plus triplicate aliquots of SCFM only, in a Tecan SPARK 10M. The incubation period was the same as for the tissue sections exposed to BODIPY-colistin to allow for comparable degradation of the signal due to time or temperature in both experimental samples and calibration samples. The best fit was calculated in DataGraph 4.5.1 (Visual Data Tools Inc.) with equal weighting for all data points and R^2 was 0.99. Raw data is supplied in Document S1.

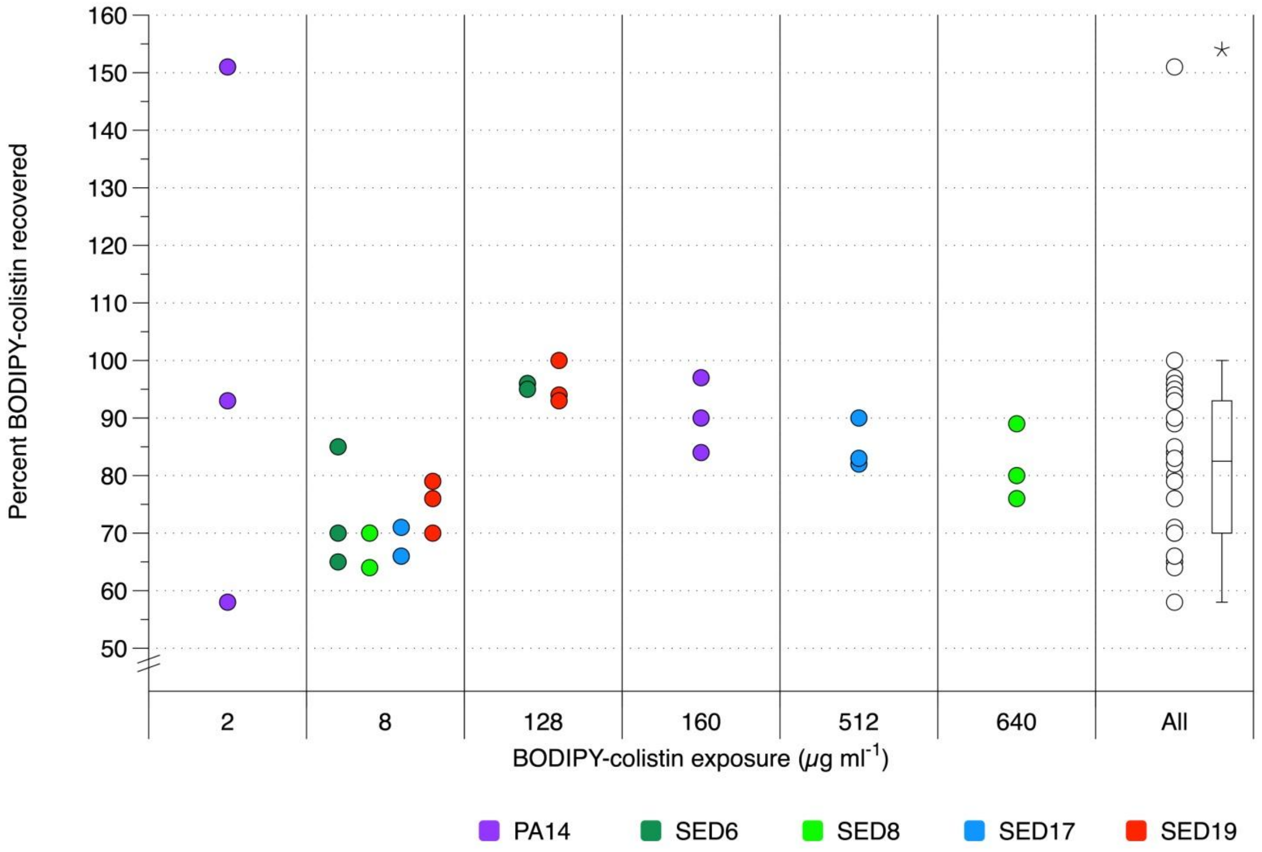

Fig. S5 Recovery of initial dose of BODIPY-colistin, as measured by fluorimetry of biofilm homogenate and surrounding SCFM after 18h exposure. Each symbol is one tissue section. Box shows 1st and 3rd quartile with median line, whiskers show interquartile range, asterisk shows outlier. Note that tissues + biofilms were exposed to BODIPY-colistin in a total volume of 1 ml SCFM, therefore concentrations correspond to total µg present. Background fluorescence from the lung tissue in the absence of either bacteria or BODIPY-colistin was very low. Raw data is supplied in Document S1.

Table S1 Reproducibility of biofilm loads on *ex vivo* tissue. The data in Figure S2 were analysed using ANOVA to test for effects of lung, strain and their interaction on bacterial load, and by linear mixed-effects models to calculate the variance in each species 'bacterial load within and between lungs. This latter information was used to calculate the intraclass correlation coefficient – this is simply the proportion of total variance explained by lung and is a commonly-used measure of repeatability. The larger this value, the greater the between-lung variance relative to the within-lung variance, i.e. a larger value means the data from replica lungs in the same treatment group are more similar, and that there is less noise present in the data due to random variation ("error"). The ICC is bounded between 0 and 1 and is conceptually similar to the commonly-used Pearson's correlation coefficient. In the table, "Interaction" records results

for the lung*strain interaction term in ANOVAs. “ICC” refers to the intra-class correlation coefficient. Raw data, R code and full results of analyses are supplied in Document S1.

	Day 2, total CFU		Day 2, CFU per mm^2		Day 7, total CFU		Day 7, CFU per mm^2	
	Interaction	ICC	Interaction	ICC	Interaction	ICC	Interaction	ICC
PAO1	$F_{2,30}$=1.42,	0.54	$F_{2,30}$=0.41,	0.33	$F_{2,30}$=0.52,	0.16	$F_{2,30}$=0.34,	0.24
PA14	p=0.26	0.73	p=0.67	0.64	p=0.60	0.42	p=0.71	0.29
